# The *Lepidopteran* KAAT1 and CAATCH1: *Orthologs* to Understand Structure–Function Relationships in Mammalian SLC6 Transporters

**DOI:** 10.1007/s11064-021-03410-1

**Published:** 2021-07-24

**Authors:** Michela Castagna, Raffaella Cinquetti, Tiziano Verri, Francesca Vacca, Matteo Giovanola, Amilcare Barca, Tiziana Romanazzi, Cristina Roseti, Alessandra Galli, Elena Bossi

**Affiliations:** 1 Department of Pharmacological and Biomolecular Sciences, Università degli Studi di Milano, Via Trentacoste 2, 20134 Milan, Italy; 2 Laboratory of Cellular and Molecular Physiology, Department of Biotechnology and Life Sciences, University of Insubria, via Dunant 3, 21100 Varese, Italy; 3 Laboratory of Applied Physiology, Department of Biological and Environmental Sciences and Technologies, University of Salento, Via Provinciale Lecce-Monteroni, 73100 Lecce, Italy; 4 Research Centre for Neuroscience, University of Insubria, Varese, Italy

**Keywords:** SLC6, Membrane transporters, Neurotransmitter transporters, Nutrient transporters, ACE2, Electrophysiology, *Xenopus laevis* oocytes

## Abstract

To the SLC6 family belong 20 human transporters that utilize the sodium electrochemical gradient to move biogenic amines, osmolytes, amino acids and related compounds into cells. They are classified into two functional groups, the Neurotransmitter transporters (NTT) and Nutrient amino acid transporters (NAT). Here we summarize how since their first cloning in 1998, the insect (*Lepidopteran*) *Orthologs* of the SLC6 family transporters have represented very important tools for investigating functional–structural relationships, mechanism of transport, ion and pH dependence and substate interaction of the mammalian (and human) counterparts.

**Supplementary Information:**

The online version contains supplementary material available at 10.1007/s11064-021-03410-1.

## Introduction

### Role of Orthologs in Translational Research

Translational research applies basic biology to understand the basis of disease and develop new therapies, medical procedures, devices, treatments and other critical medical issues [1]. A major goal in translational research is to identify the most suitable molecular, cellular and animal models for studying single physiological and/or pathological processes. Eventually, suitable models can come out by investigating distant *Orthologs* of a human gene. Differences in sequence can correlate to differences in function providing original solutions in structure–function analyses. In this respect, the increase in the number of fully sequenced genomes offers important advances in comparative genomics and translational research today [2]. *Orthologs* are genes that evolved by speciation from a common gene that retained a similar function in an ancestral species, while *Paralogs* are genes that emerged by duplication within a genome and that have often acquired a new function [3–5]. Both can be particularly adapted in structure–function studies. Over the years, the characterization of the human neurotransmitter transporters has taken advantage of the comparison of sequences and functions in phylogenetically distant *Orthologs*/*Paralogs*. Non-mammalian proteins have been used for achieving not only deep structural (bacterial *Orthologs*/*Paralogs*) but also deep functional information (invertebrate *Orthologs Paralogs*). In particular, the study of the molecular physiology of the SLC6 family members has counted on two special tools of non-human, non-vertebrate origin, i.e., the *Lepidopteran* amino acid transporters KAAT1 and CAATCH1. This review shows how studying KAAT1 and CAATCH1, two *Manduca sexta* nutrient transporters both belonging to the SLC6 family, has led to identifying many novel structural determinants strictly involved in the manifestation of major functions of the human neurotransmitter transporters.

## The SLC6 Transporter Family

**Fig. 1:**
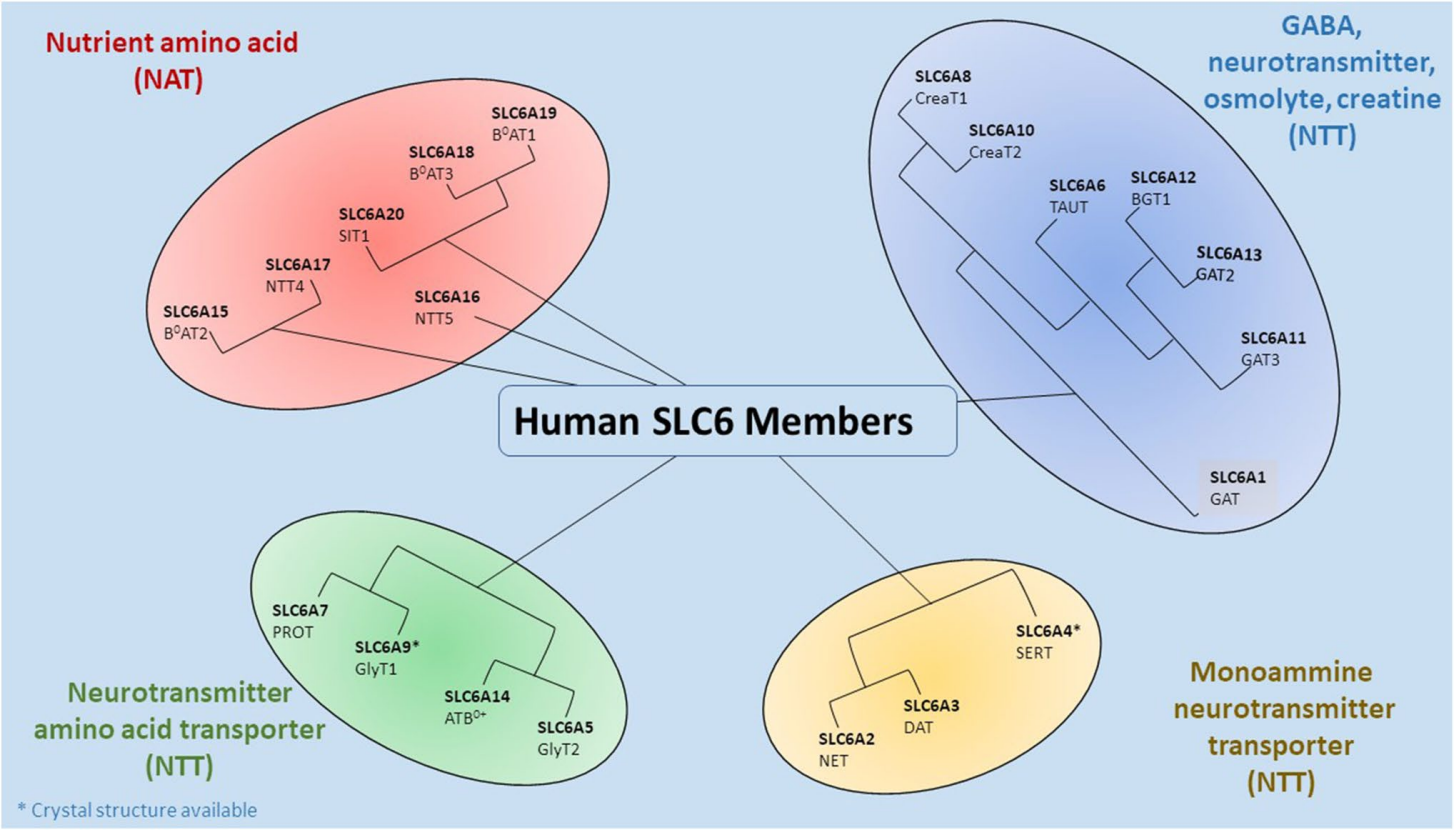
The human SLC6 transporters. For hSERT and hGlyT1 the crystal structures are available [11, 12]

The Solute carrier 6 (SLC6) [6, 7] family comprises transporters for neurotransmitters, proteinogenic amino acids, osmolytes as betaine or energy metabolites as taurine and creatine. Transporters belonging to this family are mainly involved in the regulation of neuronal communication through the reuptake of neurotransmitters and in whole-body homeostasis [8]. The family is composed of approximately 20 structurally related symporters that actively translocate amino acids and related solutes into cells against their concentration gradient using the energetically favorable coupled movement of ion(s) down their transmembrane electrochemical gradients [9]. The neurotransmitter transporters have been the first identified members giving the name of Neurotransmitter sodium symporters (NSS) or the Na^+^/Cl^−^-dependent transporters to the family [10]. This class of transporters indeed co-transport with their substrates two or three Na^+^ ions, but other ions such as Cl^−^ or K^+^ can be involved in the translocation process. Substrate affinity and sequence similarity allow division of the SLC6 family into four subgroups: GABA transporters, Monoamine transporters and Amino acid transporters (I) and (II) [8] (Fig. 1).

*The* *GABA* *transporter* *subgroup* contains transporters for GABA, betaine, taurine and creatine. GABA is the major inhibitory neurotransmitter in the brain. In humans, four distinct transporters are responsible for the transport of this neurotransmitter, namely GAT1 (SLC6A1), GAT3 (SLC6A11), BGT1 (SLC6A12), GAT2 (SLC6A13). A decrease of GABA transporters activity determines an enhancement of the inhibitory transmission due to reduced GABA reuptake after synaptic release. Consequently, GABA transporter drugs are used to treat not only seizures but also pain and anxiety [13]. This subgroup also comprises the transporters of the osmolyte taurine TauT (SLC6A6) and betaine BGT1 (SLC6A12), as well as creatine CreaT (SLC6A8), which is a storage compound for high energy phosphate bonds to replenish ATP, particularly in muscle and brain.

*The* *Monoamine* *transporter* *subgroup* contains the neurotransmitter transporters for dopamine DAT (SLC6A3), serotonin SERT (SLC6A4), and noradrenaline NET (SLC6A2). These neurotransmitters play a pivotal role in the modulation of mood and behaviour like aggression, anxiety, depression, addiction, appetite, attention etc. [14, 15]. Impairment of the function of these transporters leads to reduced clearance of monoamine transmitters, resulting in a more intense and prolonged signal. These transporters are target of the main pharmacological drugs for mood disorders and of abuse drugs [16–25]. For this neurotransmitter transporters group, the structures of a human transporter (serotonin transporter) and a *Drosophila* transporter (dopamine transporter) [11, 26, 27] are available.

*The* *Amino* *acid transporter* *subgroup* (I) includes transporters for glycine, for proline, and the general amino acid transporter ATB^0+^. Glycine is the major inhibitory neurotransmitter in the spinal cord. It is transported by two distinct proteins: the glial and the neuronal GlyT1 (SLC6A9) and GlyT2 (SLC6A5), respectively. In the last years, an increasing number of new inhibitors of these two transporters have been identified supported by data showing their beneficial effect in psychotic disease and neuropathic pain [28–31]. Moreover, in this group are present two other amino acid transporters: the proline transporter PROT (SLC6A7) [32, 33] and ATB^0+^ (SLC6A14) transporter, which is specific for neutral (0) and cationic (+) amino acids. ATB^0+^ is involved in the clearance of amino acids from secreted fluids [34] but also seems to play a role in various diseases and cancer [35–37]. For this amino acid transporter subgroup, the structure of a human transporter (glycine transporter) has recently been made available [12].

*The* *Amino* *acid* *transporter* *subgroup* (II) contains transporters responsible for amino acid homeostasis. They can be divided in turn into two groups, one including B0AT2 (SLC6A15), NTT4 (SLC6A17), and the orphan transporter NTT5 (SLC6A16) [38], and the other including SIT1 (SLC6A20), B0AT3 (SLC6A18), and B0AT1 (SLC6A19) [39]. This last involved in Hartnup disorders [40] and together with SIT1 required to be associated with ACE2 for the surface expression [41–43].

The SLC6 family transporters are widely expressed in different tissues. The neurotransmitter transporters are mainly located in the CNS. However, some of these, as NET and SERT, are also found in a subset of adrenal chromaffin cells, mast cells and blood platelets [44]. The amino acid transporters are found on the apical surface of epithelial cells, mostly in the intestine and kidney, but also in brain, lung, pituitary gland and testis. Taurine and creatine transporters are found in the brain, kidney and other non-neuronal tissues. Apart from NTT4 [45] found in vesicular compartments in the brain, all other SLC6 family transporters are located primarily in the plasma membrane. In the brain, the neurotransmitter transporters are expressed both on the presynaptic membrane to recapture released neurotransmitters and in astrocytes to remove neurotransmitters and modulate neurotransmission by a variety of mechanisms [46–49].

### Role of Chloride in SLC6

For the majority of the family members, it is widely accepted that the transport mechanism depends on the presence of chloride. In the absence of the anion, the activity of Cl^−^-dependent transporters is deeply reduced although not completely abolished (generally less than 10% of the control conditions) [50–52] but the family comprises also weakly Cl^−^- dependent (showing a 50% activity in the absence of chloride) and fully Cl^−^- independent members. Based on LeuTAa structures, a chloride binding site has been modelled for the GABA transporter GAT1 and serotonin transporter SERT [50, 51], highlighting its partial overlapping with the Na1 site for sodium that, in turns, shares some residues also with the substrate binding site, underlining how the “*Intimate contact between substrates enables transport*” [53]. Furthermore, very recently, based on *Dm*DAT structure also the chloride dependent conformational changes were defined in GlyT1 transporter [52]. These findings are supported by the observation that the independence from the anion is achieved by the presence of a negative charge (Glu290, LeuTAa numbering) (for a comprehensive observation of the findings see Fig. 5) in the position occupied by chloride in Cl^−^-dependent proteins. Mutagenesis in GAT1 [54] and the bacterial homologues TnaT and LeuTAa [55–57], and description of the chloride binding sites in DAT, SERT crystal structures [11, 27, 58], definitely confirmed the presence of the anion binding site. This corroborating the hypothesis that the halide is necessary for the neutralization of the positive charge of sodium allowing an increase in the coupling rate of the transport. GAT1 has been deeply investigated about its chloride dependence [51, 54, 59]. The modality of interaction with the anion is not clear: for GAT1 it has been demonstrated that a higher external chloride concentration favors the binding of external Na^+^ ions required for the transport process [59–63]. On the other hand, it has been also proved that the hyperpolarization of the cell membrane abolishes the dependence from the anion and renders chloride replaceable by any hydroxyl ion [62, 63]. Other evidences gathered supporting a model in which, during the normal transport cycle, chloride is not transported into the cell with the other substrates, but its association with the transporter occurs only transiently through an in/out movement of the ion from the bulk solution to the protein [60]. A recent revisitation of the substrate/ion stoichiometry of GAT1 induced transport [64] has nevertheless excluded that at physiological extracellular and intracellular chloride concentrations, a Cl^−^-independent GABA uptake may occur. In GAT1, chloride has also been shown to influence the reverse transport of the neurotransmitter [65]. This kind of transport for GAT1 was initially observed by heterologous expression [62, 63, 66]. However, a non-vesicular, calcium independent, GABA release from neurons has also been determined as a relevant component of tonic inhibition of CNS, regulating brain excitability, and has been reported in different pathological conditions including epilepsy [67, 68]. For the monoamine transporters it has been suggested that different functional states of the transporters may lead to Cl^−^ currents in native preparations whereas by heterologous expression Na^+^ uncoupled currents can be elicited [69].

## The Bacterial Homolog LeuT from *Aquifex aeolicus* (LeuT*Aa*)

Since 2005 the structure of the bacterial amino acid transporter LeuT*Aa* has been the main source of information regarding the architecture and structure-based mechanism of transport in a SLC6-type protein [19, 58, 70–72]. All these data represent a milestone to understand the functionality of different mammalian neurotransmitter transporters and model their different states [73–75]. Different conformations of LeuTAa: substrate-free state [58, 76, 77], inward-open state [58, 78–80] and competitive [81] and non-competitive inhibitor-[58] bound states [18] have revealed many mechanistic structures for the transport and transport inhibition of neurotransmitters transport proteins [72]. Only in the last years structure of the *Drosophila* dopamine transporter (dDAT) [26], the human serotonin transporter (hSERT) [11] and very recently human GlyT1 (hGlyT1) transporter [12] have been obtained, providing new and detailed crucial information for understanding the transport mechanism and the activity of inhibitors and potential drug [26, 27, 82–84].

## From KAAT1 (*Manduca sexta* Amino Acid Transporter) to GABA Transporter Structure–Function

### History - Cloning and Functional Characteristics

**Fig. 2:**
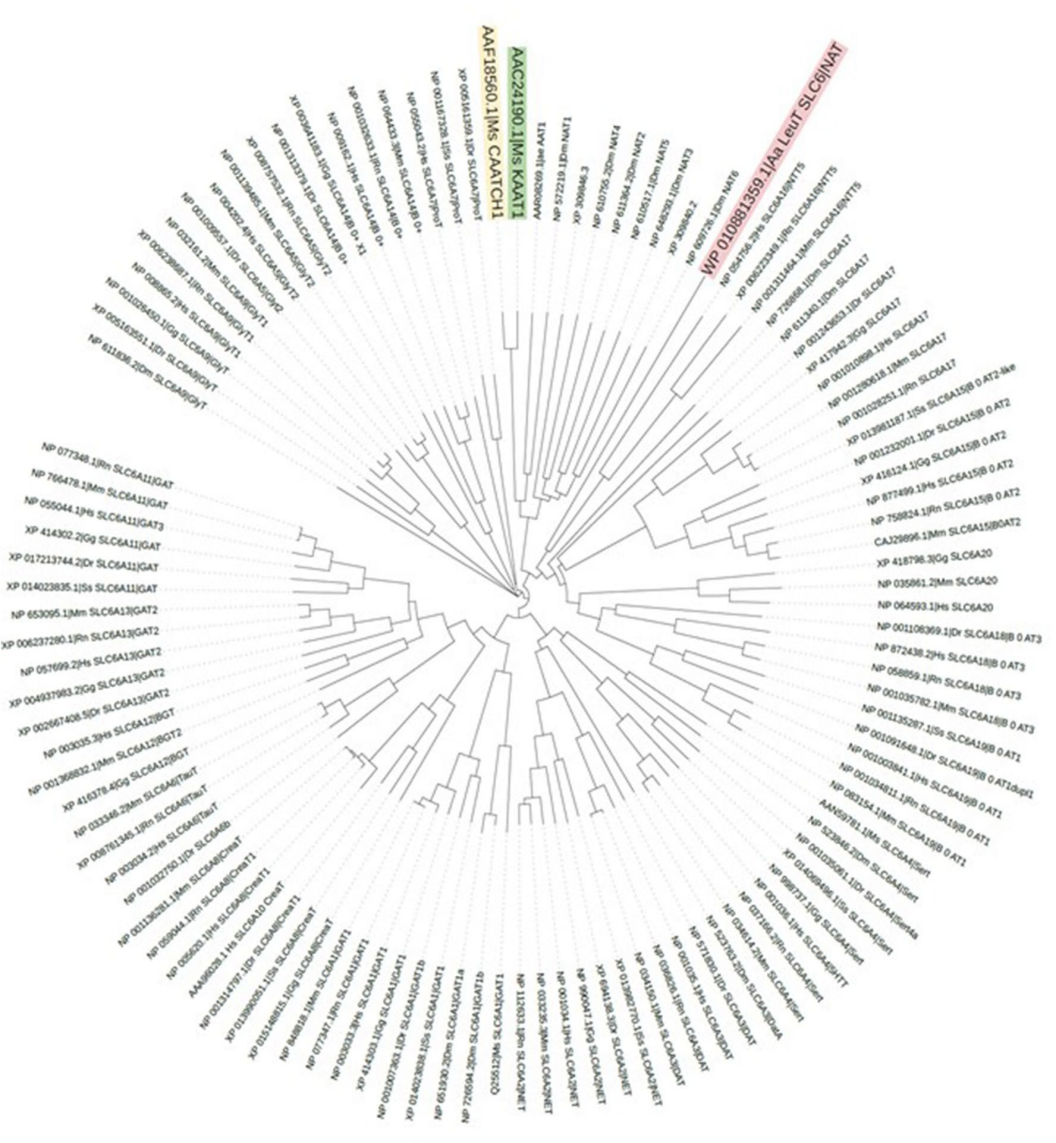
Phylogenetic relationships from selected SLC6 type transporters. Representative members of fish, insect, birds, mammalian -created with Interactive tree of life (https://itol.embl.de/) [87]

SLC6 family members are widespread from human to bacteria, and members of each subgroup are regularly found in both Protostomes and Deuterostomes (Fig. 2) suggesting a capital role in the animal cells. This review aims to underline the value of insect transporters in the study of the molecular physiology of SLC6 family members. We focus on the role of *Manduca sexta* neutral amino acid transporters KAAT1 and CAATCH1 in elucidating some fundamentals in mammalian SLC6 transporters. These two *Lepidopteran* members of the SLC6 family have been for years a unique tool for investigating the structure–function of their mammalian *Orthologs*. Their functional peculiarities can be attributed to well-defined residues, consequently permitting the identification of counterparts essential for the activity of the mammalian SLC6 members. In 2005 Boudko [85, 86] evaluating the yellow fever vector mosquito (*Aedes aegypti*) aeAAT1 homology with selected eukaryotic and prokaryotic genomic databases found more than one hundred cloned and predicted genes belonging to the SCL6 family, between them two *Manduca sexta*, that were subgrouped into the NAT cluster with other insect and mammalian transporters. Notably, the transporters of the NAT cluster appear to be specifically involved in nutrient transport processes.

**Fig. 3:**
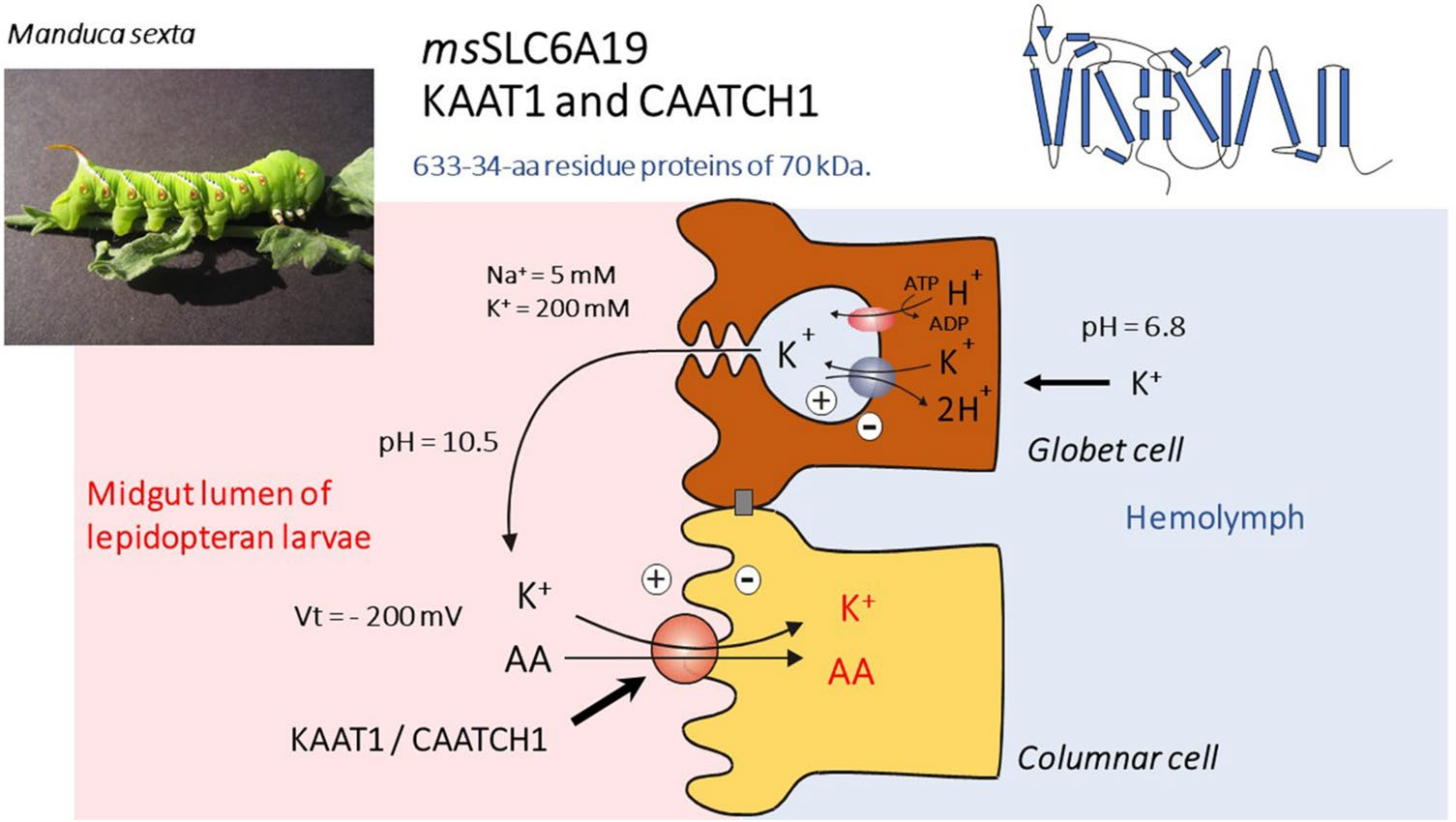
*Manduca sexta* transporters KAAT1 and CAATCH1: Structure, expression, and in vivo operating conditions

KAAT1 and CAATCH1 are K^+^-dependent amino acid transporters that have been identified in the *Lepidopteran* larvae midgut, one of the most studied tissues in *Lepidoptera* [88, 89] due to the almost unique physiological features of the transport processes taking place there [90]. The hemolymph of *Lepidopteran* larvae has a very low [Na^+^]-to-[K^+^] ratio and relatively high [Mg^2+^] and [Ca^2+^], in marked contrast to mammalian blood [91, 92]. The midgut is mainly composed of two types of cells: goblet cells and columnar cells (Fig. 3) [93, 94]. Goblet cells are involved in net secretion of K^+^. An apical electrogenic H^+^ V-ATPase in parallel with a K^+^/2H^+^ antiporter [95] are responsible for the active secretion of K^+^ from the hemolymph into the midgut lumen (Fig. 3). This “K^+^ pump” produces a transapical electrochemical potential difference for K^+^ of approximately − 200 mV, which is composed almost completely of the voltage component [91, 96–98], as well as a large pH difference between the hemolymph (pH 6.8) and the lumen (pH 10.5) since K^+^ reaches the lumen accompanied by carbonate, resulting in luminal alkalization [92]. The columnar cells are absorptive cells with a well-developed brush-border and accumulate amino acids, coupled to the cotransport of K^+^ [91, 96]. KAAT1 was the first K^+^-coupled neutral amino acid transporter cloned from a *Manduca sexta* midgut cDNA library [99]. The protein of 634 amino acids is organized in 12 TM domains, realizes the cotransport of neutral amino acids with both branched and not branched side chain in addition to glycine and, matching the condition of larval intestine, shows a maximal activity at alkaline pH values, a condition in which amino and carboxyl groups of the substrate leucine are both deprotonated [100, 101].

Sequence comparison revealed a mean sequence identity of about 35% with members of the SLC6 family but despite the intriguing homology, functional expression of KAAT1 in *Xenopus laevis* oocytes highlighted features shared with the family members and unique features that has rendered its cloning a sort of turning point in the field of secondary active transport physiology. Indeed, KAAT1 is an amino acid transporter that is activated by sodium but can accept as driver ion also potassium and lithium and shows an organic substrate specificity that is dependent on the main cation present. When Na^+^ is the driver ion, threonine is the preferred substrate and then proline, methionine and leucine with this order of preference, while when the driver is K^+^, leucine is the preferred one [102, 103]. The wide spectrum of transportable substrates matches the behaviour of most intestinal transporters and reflects the functional role of this protein in the context in which it is expressed. In the gut its main role is to accumulate as much as possible metabolites from the lumen that is, due to the vegetarian diet of the animal, normally low in amino acids and sodium content. In this scenario, the change of substrate preference according to the different driver ion suggests that the coupling properties are not due to a low ion selectivity being the protein able to modify its behaviour according to a different cation. As a further distinction from most neurotransmitter transporters of the SLC6 family, KAAT1 shows a weak chloride dependence [99, 104]. The transport activity is electrogenic: beside the classical transport associate currents, it shows pre-steady state currents and leaky currents [99, 105, 106]. The amplitude of the transport associated currents is influenced both by the driver ion and by the transported amino acid [99, 102, 103, 107]: proline, for instance, is transportable only when the membrane is highly hyperpolarized like it has been found in vivo [108]. The uncoupled currents are, as well as in other members of the family [61, 109], particularly large and show a cation selectivity sequence of Li^+^ > Na^+^  > K^+^ ≈ Rb^+^ ≈ Cs^+^ indicating that these ions interact with the protein in a specific cation binding site [106].

**Fig. 4:**
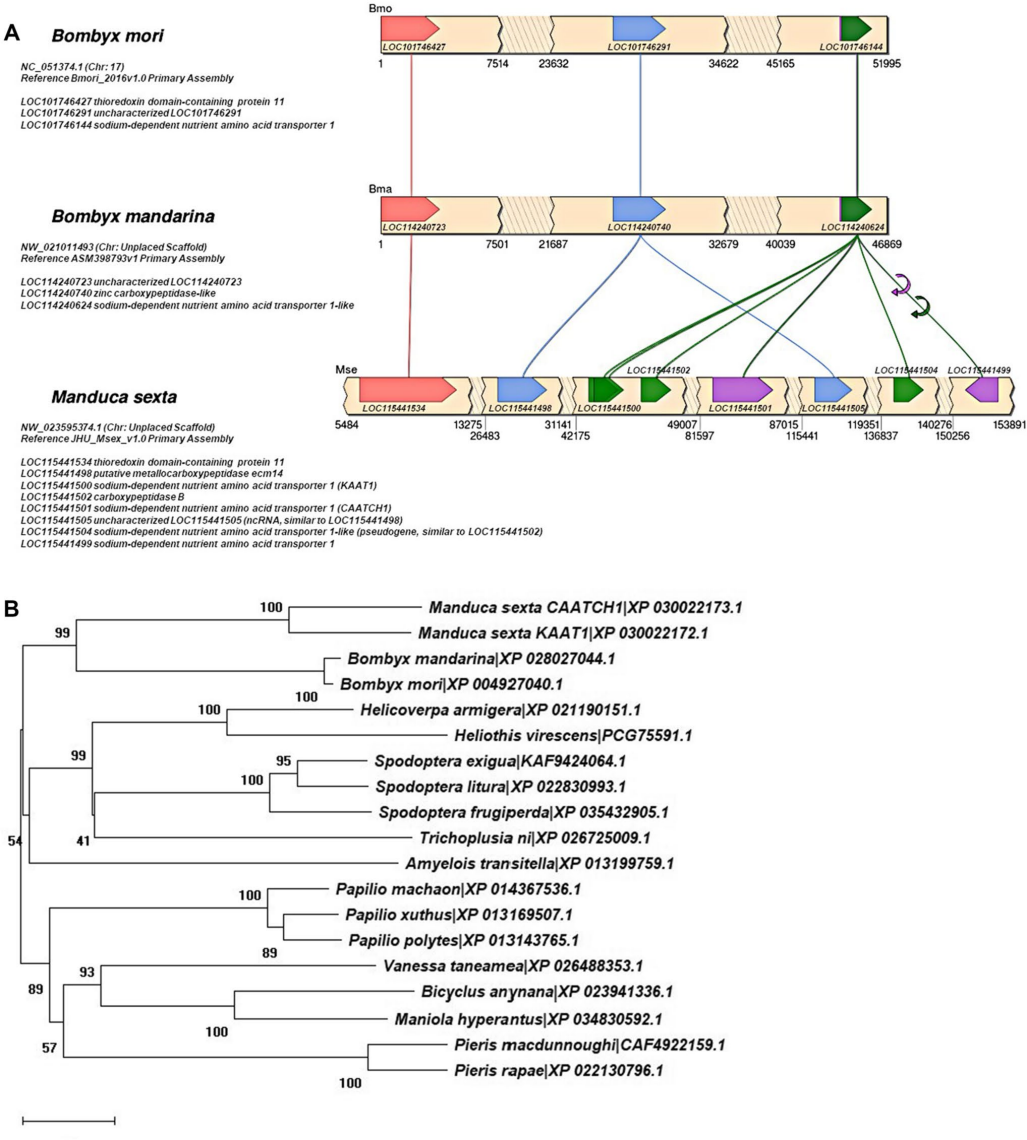
Comparison of genomic scaffolds of *Manduca* *sexta* and the closely related *Bombyx* *mori* and *Bombyx* *mandarina* genomes. **A** Large duplication event seems to be occurred in the *Manduca* *sexta* genomic region containing LOC115441498, KAAT1 (LOC11544500), LOC11544502, CAATCH1 (LOC11544501) as evident by comparison to the downstream region containing LOC11544505 and LOC11544504 genes. The region contains scattered along the scaffold other nutrient amino acid transporter genes strongly related to the SLC6 family (see e.g., LOC115441499), which suggest a more complex genomic organization of this area. **B** Phylogenetic relationships among *Lepidopteran* (moths and butterflies) sodium-dependent nutrient amino acid transporters (NATs) sharing highest similarities (67–75% identity) with *Manduca* *sexta* KAAT1/CAATCH1 transporters

From the same tissue, by a PCR-based strategy, Feldman and co-workers cloned in 2000 another transporter with a mean significant homology (37% amino acid identity) with SLC6 members and with a high degree of identity (90%) with KAAT1: the cation–anion-activated amino acid transporter/channel 1 (CAATCH1) [110]. Like KAAT1, CAATCH1 is K^+^ dependent but activated also by Na^+^, is pH-dependent and is partially chloride independent but specific features of its induced transport process distinguish it from KAAT1. CAATCH1 exhibits a peculiar behaviour of an amino acid-gated cation channel in which methionine can inhibit constitutive leakage currents whereas other substrates can activate currents [111] and shows a substrate selectivity preferring threonine in the presence of K^+^ and proline in the presence of Na^+^, whereas leucine is not accepted [103]. The differences in amino acid sequence and function of these two proteins compared with SLC6 members has represented a robust tool in the characterization of the structure–function relationships of the family. This despite the very limited genomic information available on the *Manduca sexta* genome, which had initially led to conceive complex patterns of alternative splicing of transcripts derived from a single gene, RNA editing of a single transcript or a gene duplication to account for the differences between KAAT1 and CAATCH1 To date we can reasonable assess that KAAT1 and CAATCH1 result from (a) gene duplication event(s), observable in *Manduca* *sexta* genome. KAAT1 is coded by a gene (Ref. LOC115441500) located upstream a second gene (Ref. LOC115441501), both on the (GenBan Acc. No.) NW_023595374.1 (Chr: Unplaced Scaffold) Reference JHU_Msex_v1.0 Primary Assembly (Fig. 4).

### What KAAT1 (and CAATCH1) Suggest(s) About the Structure–Function Questions of the SLC6 Family

At the beginning of the 2000s, the use of *Orthologs* was not so common in structure–function studies but KAAT1 cloned in 1998 [99] and CAATCH1 cloned in 2000 [110] showed so many interesting properties that their use in investigating structure–function of the mammalian neurotransmitter transporters became necessary. The main properties of the two transporters have thus been investigated in comparison e.g. with the well-studied mammalian GAT1 (SLC6A1). Starting from the pH modulation of the electrophysiological properties, we discovered and investigated many of the determinants involved in substrate and ion binding, chloride and pH regulation and transport steps [102–104, 112–122].

### pH Dependence

Although pH dependence varies between members of the SLC6 family and among *Orthologs* and it has never been fully investigated even between the mammalian, it represents an interesting aspect as it is hypothesized to have also a role in chloride dependence in some steps of the transport process [123]. In this review, we have decided to introduce the use of KAAT1 as a “*model orthologue*” for the study of neurotransmitter transporters of the SLC6 family starting from the first mutant of GAT1 inspired by the function of KAAT1. The first use of KAAT1 in studying GAT1 function was in 2001 [112]. In this paper, a mutation in the fifth extracellular loop of rGAT1 was produced, taking the KAAT1 sequence as a model, providing the pH dependence on the electrophysiological properties of rGAT1. The idea started from the behaviour at different pH of the rat and human serotonin transporters where a single residue was involved in the modulation of the transport current [124, 125]. The residue, before the structure of LeuT*Aa*, was considered part of 5 external loop and located after the crystallization, in the 10 TMD, proximal to the extracellular side [71]. Comparing the sequences of GAT1, rSERT, hSERT and KAAT1 in this region, the presence of positive lysine residues was considered a signature for the pH insensitive transporters. In fact, in KAAT1, which was highly pH-dependent [126], this residue is a negatively charged glutamate, as in almost insect and prokaryotic transporters [127], while it is a threonine in rSERT. GAT1 became pH-dependent after the substitution of the Lysine 448 with negatively charged glutamate. The pre-steady state current and consequently the Q/V (Charged /Voltage) relationship and the transport current were influenced by the pH and the apparent GABA affinity decreased. These findings let us in 2001 to conclude that “*The specific effects of the K448E mutation and the more general inhibition seen in the Y452E *[112]* form suggest that the extracellular loop 5 of rGAT1 may take part in forming a sort of ‘vestibule’, where Na*^+^ *ions must have access before the charge-moving transition could take place”.* Our hypothesis was confirmed by the LeuT*Aa* structure, where the residues close to the extracellular side of the TMD10 form together with the EL4 and residues of the TMD1 the extracellular gate. Almost ten years later, the same residue was discovered to be also involved in the interaction of tricyclic antidepressants with GAT1, confirming its role in the extracellular gate [128].

### Ionic Dependence

**Fig. 5:**
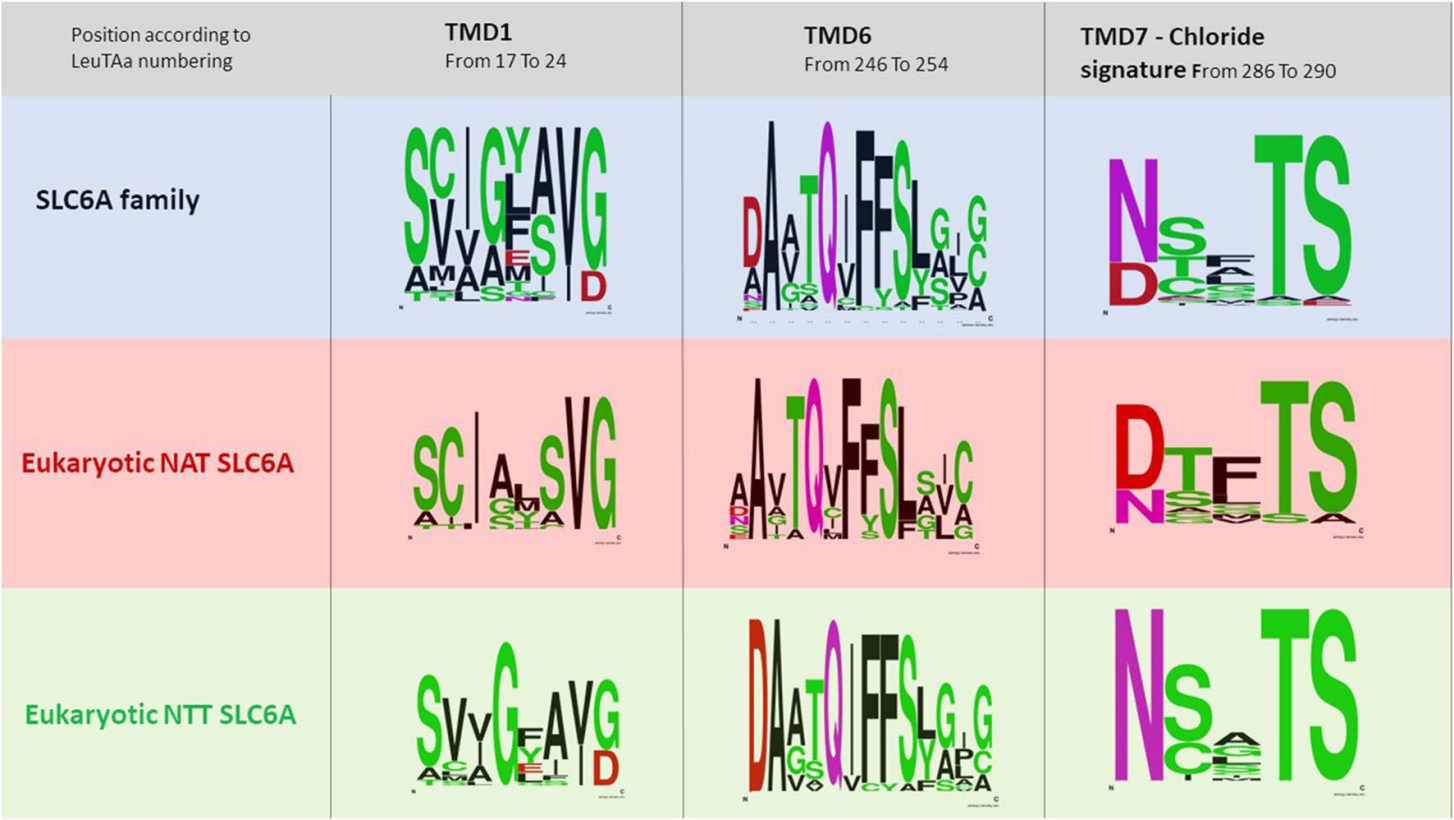
WebLogo of the indicated functional regions. The amino acid sequence considered are indicated and referred to LeuT*Aa* numbering [137]

When, in 2007, Kanner [51] and Forrest [50] proposed the chloride binding site from the asparagine N286 to glutamate E290, LeuT*Aa* numbering (Fig. 5), we looked with interest to the first amino acid of the series whose function was characterized in 2004 [102] for KAAT1. D338 mutants strongly affected ionic dependence. In our work, the D338 residue was identified as a potential actor because it was present only in the K^+^-accepting cotransporters KAAT1 and CAATCH1. Aspartate was mutated in the corresponding asparagine present in rGAT1, rSERT and hDAT and other neutral or conservative residues. Mutants D338G and D338C of KAAT1 led to non-functional transporters, instead D338E and D338N, conservative and semiconservative mutants, displayed altered ionic selectivity in electrophysiological behaviour and uptake experiments, pointing to a role of D338 position in K^+^ interaction and in coupling of amino acid and cation fluxes. Interestingly, our conclusions anticipated one of the most important novelties revealed by LeuT*Aa*’s crystal structure: i.e., that the thermodynamic coupling of ion and substrate transport is achieved utilizing the spatial proximity and direct interaction of the binding sites, as the corresponding residue N286 in LeuT*Aa* was found to be part of the Na1 binding site.. Noteworthy some years later we hypothesized that the D338 negative charge could be the player involved in the ability of KAAT1 and CAATCH1 to work in weakly chloride-dependent mode [104]. By a second-site suppressor approach, the altered uptake observed in D338E mutant could be recovered by the simultaneous substitution of the specific Lys 102 with Val. The same behaviour was observed in the corresponding mutant (V56K-N264D) of the bacterial tryptophan transporter TnaT from *Symbiobacterium thermophilum,* thus giving a functional meaning to the structural proximity observed between TMD2 and TMD7 in the LeuTAa 3D structure [116]. A direct interaction between these residues was proved by the thiol crosslinking inhibition observed in the D338C/K102C mutant, and considering that in LeuT*Aa* N286 participates in the coordination of sodium in the Na1 binding site, our analysis outlined as during the transport cycle Lys102, through its positive charge, may maintain D338E in position, maximizing the coordination of Na^+^ and K^+^ and facilitating cation-substrate coupling. In KAAT1 and CAATCH1 chloride differently influenced the translocation according to the organic substrate and the driving ion, affecting the ion coupling. The uptake was always reduced by the complete absence of chloride but the transport currents if the substrate was leucine in sodium or threonine in potassium resulted completely independent of the presence of Cl^−^ ions. Focusing on the region where TM1 adopts an extended conformation connecting TM1a and TM1b, i.e. the putative Na^+^ and leucine binding site, it stands out that the three amino acids are specific for KAAT1 and CAATCH1. The threonine 67 (N21 in LeuT*Aa*) involved in substrate binding located between the putative Na1 and Na2 binding site (Fig. 4), is present only in KAAT1 and CAATCH1 and the sodium binding sites in KAAT1 (and CAATCH1) are not conserved: Ala66 corresponds to Gly20 in the Na2 site of LeuT*Aa*, and Ser68, corresponds to Ala22 in the Na1 site. Mutations of KAAT1 and CAATCH1 site in this position affected the sodium dependence and altered the chloride requirement suggesting a role in the coupling step of the transport process [59, 118, 119, 121, 129]. A clearer view of the role of chloride in SLC6 was offered by the comparative characterization of some orthologues of B0AT1 (SLC6A19) and B0AT2 (SLC6A15) [130–133]. These nutrient transporters of the SLC6 family, in contrast to other members, are completely chloride-independent and in their sequence in the chloride binding site asparagine is in most cases substituted by aspartate. As stated above, other members of the family share with KAAT1 and CAATCH1 the weak chloride dependence [134–136] and, interestingly, all of them are responsible for the uptake of amino acids at the intestinal and renal epithelial cells even if they are also expressed in internal districts. Thus, the weak chloride dependence (or the complete chloride independence of some insect eukaryotic members [86] and all prokaryotic proteins) seems to be linked to the facing of the protein to the extracellular environment.

We can speculate that prokaryotic transporters did not evolve the dependence from chloride being these proteins counteracting an environment not always constant in its composition, in which the dependence from the availability of another ion, apart from sodium that is necessary for the activation of the transport, could be a disadvantage in terms of competition for nutrients. The solution was found in keeping the required negative charge constantly associated with the transporter (and varying its protonation state according to the different step of the transport cycle) [51]. Evolution probably allowed the loosing of this adaptation in mammalian transporters being these proteins harboured in a system that steadily bath cells membrane with a fluid in which the chloride concentration is never limiting. Still open is the question regarding the evolutionary advantage gained by these molecules in acquiring the dependence from this anion (or, in other terms, why they have lost the negative charge present in their prokaryotic ancestor). More complicated is the case of proteins that face the extracellular environment in eukaryotic organisms that have evolved a similar or identical structure, in the putative chloride binding site, to those of mammalian strictly chloride dependent proteins but also show behaviour that, somehow, is superimposable to that of bacterial members of the family. We can again speculate that this could be an evolutionary adaptation. It is easy to think of a common origin for eukaryotic SLC6 members, those that are active in apical membranes of renal or intestinal epithelia are devoted to uptake their substrates in conditions not highly controlled as found by their homologues in the internal environment of the organisms. The loosing of chloride dependence could be a sort of exit strategy to make the uptake less susceptible to variation of the environmental conditions. For some of these mammalian chloride-independent proteins, the absence of the anion does not modify the uptake capacity whereas for insect amino acid transporters it reduces the activity up to 50% according to the transported substrate, indicating a more complex role for the halide in these transporters. Our speculations seem to be at least in part sustained by the fact that both KAAT1 and CAATCH1 in the position corresponding to Glu290 of LeuTAa bear a neutral polar residue, as found in Cl^−^-dependent carriers, but possess as residue bridging the two Na^+^ binding sites, Thr67, that is not conserved in the family, is essential for the functional coupling of substrates flux and which modification influences the interaction with chloride [121], highlighting as in these insect transporters the interaction with the anion is different from that occurring in full chloride dependent transporters.

### Residues Involved in Na/K and Substrate Binding

As reported above often residues involved in chloride dependence, when mutated, also modified the ionic coupling, underling that the anions and cations cooperate in the translocation process. In addition to the residues already mentioned, other functional determinants have been identified by mutation using KAAT1 and CAATCH1 as models. Most of these amino acids residues have been identified by utilizing the comparison between the two sequences of CAATCH1 and KAAT1. The two *Manduca sexta* transporters not only differ from the mammalian members of the SLC6 family for their ability to use K^+^ as driving ion but also differ from one another in terms of substrate selectivity and a very small number of residues can be responsible for the differences. The two proteins have sequence identity of 90.15%, that is to say, that they differ for only 63 amino acids and that between them only 41 are located in the central regions [103]. KAAT1 and CAATCH1 mutants, chimaera proteins and chained constructs, concatemers, studied over the years, have often anticipated findings that have been confirmed by the publication of the structures of the SLC6 members [11, 27, 71]. Among these, in 2004 the construction of chimeric proteins between the two transporters had predicted the presence of a central region fundamental for the selectivity to substrates [103]. The apparent discrepancy between the results reported in the paper that identifies TMDs 4 to 8 as responsible for selectivity is easily explained by underlining the full sequence identity between the two *Manduca* proteins in TMDs 1 and 3. Taking advantage of the fact that CAATCH1 and KAAT1 give rise to specific kinds of current depending on the cotransporter ion, pH, transported amino acid and membrane voltage, in 2007 we have created concatemered proteins consisting of one KAAT1 protein covalently linked to another KAAT1 (K–K concatamer) or CAATCH1 (K–C concatamer) and vice versa (C–C concatamer and C–K concatamer). Studying the electrophysiological properties of these constructs we demonstrated that their activity was compatible with a monomeric functional protein. Nevertheless, the FRET studies in the same work suggested that these proteins form oligomers and confirm that, when expressed on the plasma membrane the COOH termini of the protein was adjacent to the other transporters subunits [115] in agreement with the atomic structure of LeuT*Aa* and data from other SLC6 members [138–142].

Another residue involved in substrate interaction was identified using the LeuT*Aa* structure as a working model. It is Ser308 involved in substrate binding located in non-helix region of the TMD6 (Fig. 5). This residue is a threonine in CAATCH1. The electrophysiological data collected on the two *Manduca* mutants showed that serine has the main role in substrate selectivity and its transfer into the cytoplasmic environment [117]. Our results were comparable to a paper published at the same time presenting similar results that explain the different selectivity of GlyT1 and 2 [143]. A further feature of CAATCH1 and KAAT1 that differentiates them from vertebrate counterparts is the ability of these two transporters to transport D-amino acids [120]. To our knowledge, only one other member of the SLC6 family was functionally demonstrated to be capable of promoting the translocation of these substrates [144]. The data are interesting not only for the result itself but for the other two aspects. First, the electrophysiological characteristics highlighted by the transport, in particular by competition experiments that allowed us to hypothesize the existence of a second binding site for the substrate with a lower affinity compared to the main one, fundamental for the selectivity of the transporters. Furthermore, in this work, the presence of substrates normally not present in the cytoplasmatic environment, allowed us to develop an alternative method for investigating the actual translocation of the substrate inside the cell, we evaluated the amount of D-amino acids in the single oocyte by analyzing the cytoplasmic content by HPLC [120]. This method has only recently been implemented by the use of GS-MS allowing to measure in a determined and precise way the basal content of metabolite in oocytes and, in oocytes expressing different membrane transporters, the amount of transported substrate in a single cell after exposure to different medium [145]. Finally, it is important to underline how the transport of D-amino acids by the insect NATs could be of particular interest, given the recent approach that involves the use of insects for the treatment of organic waste for the production of molecules of nutraceutical, pharmacological and nutritional interest [146, 147].

### KAAT1, CAATCH1 and Other SLC6 Nutrient Transporters from Insect to Human - Alone or with Ancillary Protein

Finally, the insects nutrient transporters are expressed on the plasma membrane even in the absence of the ancillary proteins, requested by the vertebrates and fish counterparts [42, 43, 148–150]. The ability to be expressed or not in the plasma membrane was investigated with different approaches in different species. This has revealed a complex picture typical of the NAT group of transporters. Some proteins need the ancillary protein to reach the membrane, other whose expression was instead greatly increased by the presence of ACE2 or collectrin [149, 150]. Only recently, due to the attention paid to ACE2 as a receptor for SARS-COV2, the three-dimensional structure that shows the interaction between NATs and the accessory protein became available and the story summarized [41]. The ancillary proteins have an important role in dimerization that is needed for membrane localization. The structure of B^0^AT1 with ACE2 allowed identification of the residues of the transporter involved in the interaction [151]. In this scenario. KAAT1 and CAATCH1 can be useful tools for investigating the possible role of the oligomerization of nutrient transporters in membrane localization. Different indications suggest the possible access of the coronavirus from the compartments open to the external environment, like the gut lumen. The necessity to be expressed together with NAT transporters causes the accessory protein ACE2 to be widely present as well. The role of ACE2 in interacting with coronaviruses has been highlighted since 2002 [152]. The copious presence at the brush border of the small intestine of ACE2 and of another aminopeptidase N [153, 154], that is also a coronavirus receptor and ancillary protein for NAT transporter, and the presence of the virus in patients samples, indicate the possibility of faecal-oral transmission. With this in mind, the future aim is to use the *Orthologs* model to understand the interaction between NAT and ancillary proteins to better understand these relations and possibly to give our contribution in developing new drugs for fighting the infection.

## Conclusions

Differences in sequence correlate with differences in function in membrane transporters and defining the role(s) of either single residues or functional (amino acid) regions help understaning the translocation process(es) in these proteins more and more often recognized to be involved in health and disease statuses. What we have reported here summarizes part of the knowledge acquired over the last two decades on the SLC6 transporters, mainly using two tools: distant orthologues and electrophysiological approaches. Although not amongst the most commonly used model organisms, *Manduca* *sexta* has certainly revealed its complementarity to the study of the SLC6 transporters, sometimes anticipating observation later confirmed and reinforced in the mammalian (and human) model. What we have learned in these 20 years has been above all the importance of the differences. In recent years, especially with increasing the translational medicine approaches, it becomes progressively important to identify the appropriate model for studying a specific cellular process. Furthermore, considering the growing importance of transporters, and of neurotransmitter transporters as targets of new and more specific drugs, it will be significant not only to exploit known animal models but also to focus on those ‘non-conventional’ model organisms that fit the specific process to be studied. For this reason, a comparative functional approach in characterizing new and old proteins will be ever more critical to valuably support the translational approach, thus leading ‘non-medical’ basic biology to increasingly support the new discoveries in medicine, health, and welfare aspect of everyday life.

## Supplementary Information

Below is the link to the electronic supplementary material.

Supplementary file1 (XLSX 19 kb)

## Data Availability

All data are available in the previous published work and as supplement materials.

## References

[1] Woolf SH (2008). The meaning of translational research and why it matters. JAMA.

[2] Nichio BTL, Marchaukoski JN, Raittz RT (2017). New tools in orthology analysis: a brief review of promising perspectives. Front Genet.

[3] Fox JG, Bennett BT, Fox JG, Anderson LC, Otto GM, Pritchett-Corning KR, Whary MT (2015). Chapter 1 - Laboratory animal medicine: historical perspectives. Laboratory animal medicine.

[4] Lobert VH, Mouradov D, Heath JK (2016). Focusing the spotlight on the zebrafish intestine to illuminate mechanisms of colorectal cancer. Adv Exp Med Biol.

[5] Khan KM, Collier AD, Meshalkina DA, Kysil EV, Khatsko SL, Kolesnikova T, Morzherin YY, Warnick JE, Kalueff AV, Echevarria DJ (2017). Zebrafish models in neuropsychopharmacology and CNS drug discovery. Br J Pharmacol.

[6] Hediger MA, Romero MF, Peng JB, Rolfs A, Takanaga H, Bruford EA (2004). The ABCs of solute carriers: physiological, pathological and therapeutic implications of human membrane transport proteins - Introduction. Pflugers Archiv-Eur J Physiol.

[7] Hediger MA, Clemencon B, Burrier RE, Bruford EA (2013). The ABCs of membrane transporters in health and disease (SLC series): introduction. Mol Aspects Med.

[8] Broer S, Gether U (2012). The solute carrier six family of transporters. Br J Pharmacol.

[9] Chen NH, Reith ME, Quick MW (2004). Synaptic uptake and beyond: the sodium- and chloride-dependent neurotransmitter transporter family SLC6. Pflugers Arch.

[10] Nelson N (1998). The family of Na^+^/Cl^−^ neurotransmitter transporters. J Neurochem.

[11] Coleman JA, Green EM, Gouaux E (2016). X-ray structures and mechanism of the human serotonin transporter. Nature.

[12] Shahsavar A, Stohler P, Bourenkov G, Zimmermann I, Siegrist M, Guba W, Pinard E, Sinning S, Seeger MA, Schneider TR, Dawson RJP, Nissen P (2021). Structural insights into the inhibition of glycine reuptake. Nature.

[13] Clausen RP, Madsen K, Larsson OM, Frolund B, Krogsgaard-Larsen P, Schousboe A (2006). Structure-activity relationship and pharmacology of Gamma-aminobutyric acid (GABA) transport inhibitors. Adv Pharmacol.

[14] Ramamoorthy S, Shippenberg TS, Jayanthi LD (2011). Regulation of monoamine transporters: role of transporter phosphorylation. Pharmacol Ther.

[15] Hahn MK, Blakely RD (2007). The functional impact of SLC6 transporter genetic variation. Annu Rev Pharmacol Toxicol.

[16] Lester HA, Cao Y, Mager S (1996). Listening to neurotransmitter transporters. Neuron.

[17] Andersen J, Ladefoged LK, Wang D, Kristensen TN, Bang-Andersen B, Kristensen AS, Schiott B, Stromgaard K (2015). Binding of the multimodal antidepressant drug vortioxetine to the human serotonin transporter. ACS Chem Neurosci.

[18] Singh SK, Yamashita A, Gouaux E (2007). Antidepressant binding site in a bacterial homologue of neurotransmitter transporters. Nature.

[19] Rudnick G (2007). What is an antidepressant binding site doing in a bacterial transporter?. ACS Chem Biol.

[20] Cheng MH, Bahar I (2019). Monoamine transporters: structure, intrinsic dynamics and allosteric regulation. Nat Struct Mol Biol.

[21] Kahlig KM, Javitch JA, Galli A (2004). Amphetamine regulation of dopamine transport. Combined measurements of transporter currents and transporter imaging support the endocytosis of an active carrier. J Biol Chem.

[22] Buchmayer F, Schicker K, Steinkellner T, Geier P, Stubiger G, Hamilton PJ, Jurik A, Stockner T, Yang JW, Montgomery T, Holy M, Hofmaier T, Kudlacek O, Matthies HJ, Ecker GF, Bochkov V, Galli A, Boehm S, Sitte HH (2013). Amphetamine actions at the serotonin transporter rely on the availability of phosphatidylinositol-4,5-bisphosphate. Proc Natl Acad Sci U S A.

[23] Daws LC, Avison MJ, Robertson SD, Niswender KD, Galli A, Saunders C (2011). Insulin signaling and addiction. Neuropharmacology.

[24] Kahlig KM, Binda F, Khoshbouei H, Blakely RD, McMahon DG, Javitch JA, Galli A (2005). Amphetamine induces dopamine efflux through a dopamine transporter channel. Proc Natl Acad Sci U S A.

[25] Shekar A, Aguilar JI, Galli G, Cozzi NV, Brandt SD, Ruoho AE, Baumann MH, Matthies HJG, Galli A (2017). Atypical dopamine efflux caused by 3,4-methylenedioxypyrovalerone (MDPV) via the human dopamine transporter. J Chem Neuroanat.

[26] Penmatsa A, Wang KH, Gouaux E (2013). X-ray structure of dopamine transporter elucidates antidepressant mechanism. Nature.

[27] Penmatsa A, Wang KH, Gouaux E (2015). X-ray structures of drosophila dopamine transporter in complex with nisoxetine and reboxetine. Nat Struct Mol Biol.

[28] Aragon C, Lopez-Corcuera B (2005). Glycine transporters: crucial roles of pharmacological interest revealed by gene deletion. Trends Pharmacol Sci.

[29] Javitt DC (2012). Glycine transport inhibitors in the treatment of schizophrenia. Handb Exp Pharmacol.

[30] Vandenberg RJ, Ryan RM, Carland JE, Imlach WL, Christie MJ (2014). Glycine transport inhibitors for the treatment of pain. Trends Pharmacol Sci.

[31] Vandenberg RJ, Mostyn SN, Carland JE, Ryan RM (2016). Glycine transporter2 inhibitors: getting the balance right. Neurochem Int.

[32] Schulz D, Morschel J, Schuster S, Eulenburg V, Gomeza J (2018). Inactivation of the mouse L-proline transporter PROT alters glutamatergic synapse biochemistry and perturbs behaviors required to respond to environmental changes. Front Mol Neurosci.

[33] Velaz-Faircloth M, Guadaño-Ferraz A, Henzi VA, Fremeau RT (1995). Mammalian brain-specific L-proline transporter. Neuronal localization of mRNA and enrichment of transporter protein in synaptic plasma membranes. J Biol Chem.

[34] Sikder MOF, Yang S, Ganapathy V, Bhutia YD (2017). The Na^+^/Cl^−^-coupled, broad-specific, amino acid transporter SLC6A14 (ATB(0,+)): emerging roles in multiple diseases and therapeutic potential for treatment and diagnosis. AAPS J.

[35] Coothankandaswamy V, Cao S, Xu Y, Prasad PD, Singh PK, Reynolds CP, Yang S, Ogura J, Ganapathy V, Bhutia YD (2016). Amino acid transporter SLC6A14 is a novel and effective drug target for pancreatic cancer. Br J Pharmacol.

[36] Bhutia YD, Ganapathy V (2016). Glutamine transporters in mammalian cells and their functions in physiology and cancer. Biochim Biophys Acta.

[37] Bhutia YD, Babu E, Prasad PD, Ganapathy V (2014). The amino acid transporter SLC6A14 in cancer and its potential use in chemotherapy. Asian J Pharm Sci.

[38] Farmer MK, Robbins MJ, Medhurst AD, Campbell DA, Ellington K, Duckworth M, Brown AM, Middlemiss DN, Price GW, Pangalos MN (2000). Cloning and characterization of human NTT5 and v7–3: two orphan transporters of the Na^+^/Cl^−^ -dependent neurotransmitter transporter gene family. Genomics.

[39] Broer S (2006). The SLC6 orphans are forming a family of amino acid transporters. Neurochem Int.

[40] Kleta R, Romeo E, Ristic Z, Ohura T, Stuart C, Arcos-Burgos M, Dave MH, Wagner CA, Camargo SR, Inoue S, Matsuura N, Helip-Wooley A, Bockenhauer D, Warth R, Bernardini I, Visser G, Eggermann T, Lee P, Chairoungdua A, Jutabha P, Babu E, Nilwarangkoon S, Anzai N, Kanai Y, Verrey F, Gahl WA, Koizumi A (2004). Mutations in SLC6A19, encoding B0AT1, cause Hartnup disorder. Nat Genet.

[41] Camargo SMR, Vuille-Dit-Bille RN, Meier CF, Verrey F (2020). ACE2 and gut amino acid transport. Clin Sci (Lond).

[42] Singer D, Camargo SM (2011). Collectrin and ACE2 in renal and intestinal amino acid transport. Channels (Austin).

[43] Danilczyk U, Sarao R, Remy C, Benabbas C, Stange G, Richter A, Arya S, Pospisilik JA, Singer D, Camargo SM, Makrides V, Ramadan T, Verrey F, Wagner CA, Penninger JM (2006). Essential role for collectrin in renal amino acid transport. Nature.

[44] Kristensen AS, Andersen J, Jorgensen TN, Sorensen L, Eriksen J, Loland CJ, Stromgaard K, Gether U (2011). SLC6 neurotransmitter transporters: structure, function, and regulation. Pharmacol Rev.

[45] Parra LA, Baust T, El Mestikawy S, Quiroz M, Hoffman B, Haflett JM, Yao JK, Torres GE (2008). The orphan transporter Rxt1/NTT4 (SLC6A17) functions as a synaptic vesicle amino acid transporter selective for proline, glycine, leucine, and alanine. Mol Pharmacol.

[46] Hamilton NB, Attwell D (2010). Do astrocytes really exocytose neurotransmitters?. Nat Rev Neurosci.

[47] Lie MEK, Al-Khawaja A, Damgaard M, Haugaard AS, Schousboe A, Clarkson AN, Wellendorph P (2017). Glial GABA transporters as modulators of inhibitory signalling in epilepsy and stroke. Adv Neurobiol.

[48] Ghirardini E, Wadle SL, Augustin V, Becker J, Brill S, Hammerich J, Seifert G, Stephan J (2018). Expression of functional inhibitory neurotransmitter transporters GlyT1, GAT-1, and GAT-3 by astrocytes of inferior colliculus and hippocampus. Mol Brain.

[49] Gadea A, López-Colomé AM (2001). Glial transporters for glutamate, glycine, and GABA: II. GABA transporters. J Neurosci Res.

[50] Forrest LR, Tavoulari S, Zhang YW, Rudnick G, Honig B (2007). Identification of a chloride ion binding site in Na^+^/Cl^−^ dependent transporters. Proc Natl Acad Sci U S A.

[51] Zomot E, Bendahan A, Quick M, Zhao Y, Javitch JA, Kanner BI (2007). Mechanism of chloride interaction with neurotransmitter: sodium symporters. Nature.

[52] Zhang YW, Uchendu S, Leone V, Bradshaw RT, Sangwa N, Forrest LR, Rudnick G (2021). Chloride-dependent conformational changes in the GlyT1 glycine transporter. Proc Natl Acad Sci U S A.

[53] Kanner BI (2005). Molecular physiology: intimate contact enables transport. Nature.

[54] Ben Yona A, Bendahan A, Kanner BI (2010). A glutamine residue conserved in the neurotransmitter: sodium: symporters is essential for the interaction of chloride with the GABA transporter GAT-1. J Biol Chem.

[55] Tavoulari S, Rizwan AN, Forrest LR, Rudnick G (2011). Reconstructing a chloride-binding site in a bacterial neurotransmitter transporter homologue. J Biol Chem.

[56] Tavoulari S, Margheritis E, Nagarajan A, DeWitt DC, Zhang YW, Rosado E, Ravera S, Rhoades E, Forrest LR, Rudnick G (2016). Two Na^+^ sites control conformational change in a neurotransmitter transporter homolog. J Biol Chem.

[57] Kantcheva AK, Quick M, Shi L, Winther AM, Stolzenberg S, Weinstein H, Javitch JA, Nissen P (2013). Chloride binding site of neurotransmitter sodium symporters. Proc Natl Acad Sci U S A.

[58] Penmatsa A, Gouaux E (2013). How LeuT shapes our understanding of the mechanisms of sodium-coupled neurotransmitter transporters. J Physiol.

[59] Giovannardi S, Fesce R, Bossi E, Binda F, Peres A (2003). Cl- affects the function of the GABA cotransporter rGAT1 but preserves the mutal relationship between transient and transport currents. Cell Mol Life Sci.

[60] Cherubino F, Bertram S, Bossi E, Peres A (2012). Pre-steady-state and reverse transport currents in the GABA transporter GAT1. Am J Physiol Cell Physiol.

[61] Mager S, KleinbergerDoron N, Keshet GI, Davidson N, Kanner BI, Lester HA (1996). Ion binding and permeation at the GABA transporter GAT1. J Neurosci.

[62] Lu CC, Hilgemann DW (1999). GAT1 (GABA: Na^+^:Cl^−^) cotransport function. Kinetic studies in giant Xenopus oocyte membrane patches. J Gen Physiol.

[63] Lu CC, Hilgemann DW (1999). GAT1 (GABA:Na^+^:Cl^−^) cotransport function. Steady state studies in giant Xenopus oocyte membrane patches. J Gen Physiol.

[64] Willford SL, Anderson CM, Spencer SR, Eskandari S (2015). Evidence for a revised ion/substrate coupling stoichiometry of GABA transporters. J Membr Biol.

[65] Bertram S, Cherubino F, Bossi E, Castagna M, Peres A (2011). Gaba reverse transport by the neuronal cotransporter gat1: influence of internal chloride depletion. Am J Physiol Cell Physiol.

[66] Cammack JN, Rakhilin SV, Schwartz EA (1994). A GABA transporter operates asymmetrically and with variable stoichiometry. Neuron.

[67] Richerson GB, Wu Y (2004). Role of the GABA transporter in epilepsy. Adv Exp Med Biol.

[68] Richerson GB, Wu Y (2003). Dynamic equilibrium of neurotransmitter transporter not just for reuptake anymore. J Neurophysiol.

[69] De Felice LJ (2016). Chloride requirement for monoamine transporters. Pflugers Arch.

[70] Henry LK, Meiler J, Blakely RD (2007). Bound to be different: neurotransmitter transporters meet their bacterial cousins. Mol Interv.

[71] Yamashita A, Singh SK, Kawate T, Jin Y, Gouaux E (2005). Crystal structure of a bacterial homologue of Na^+^/Cl^–^ dependent neurotransmitter transporters. Nature.

[72] Joseph D, Pidathala S, Mallela AK, Penmatsa A (2019). Structure and gating dynamics of Na(+)/Cl(−) coupled neurotransmitter transporters. Front Mol Biosci.

[73] Sohail A, Jayaraman K, Venkatesan S, Gotfryd K, Daerr M, Gether U, Loland CJ, Wanner KT, Freissmuth M, Sitte HH, Sandtner W, Stockner T (2016). The environment shapes the inner vestibule of LeuT. PLoS Comput Biol.

[74] Khan JA, Sohail A, Jayaraman K, Szöllősi D, Sandtner W, Sitte HH, Stockner T (2020). The amino terminus of LeuT changes conformation in an environment sensitive manner. Neurochem Res.

[75] Grouleff J, Koldsø H, Miao Y, Schiøtt B (2017). Ligand binding in the extracellular vestibule of the neurotransmitter transporter homologue LeuT. ACS Chem Neurosci.

[76] Malinauskaite L, Said S, Sahin C, Grouleff J, Shahsavar A, Bjerregaard H, Noer P, Severinsen K, Boesen T, Schiott B, Sinning S, Nissen P (2016). A conserved leucine occupies the empty substrate site of LeuT in the Na(+)-free return state. Nat Commun.

[77] Krishnamurthy H, Gouaux E (2012). X-ray structures of LeuT in substrate-free outward-open and apo inward-open states. Nature.

[78] Zhao C, Stolzenberg S, Gracia L, Weinstein H, Noskov S, Shi L (2012). Ion-controlled conformational dynamics in the outward-open transition from an occluded state of LeuT. Biophys J.

[79] Stolzenberg S, Li Z, Quick M, Malinauskaite L, Nissen P, Weinstein H, Javitch JA, Shi L (2017). The role of transmembrane segment 5 (TM5) in Na_2_ release and the conformational transition of neurotransmitter: sodium symporters toward the inward-open state. J Biol Chem.

[80] Terry DS, Kolster RA, Quick M, LeVine MV, Khelashvili G, Zhou Z, Weinstein H, Javitch JA, Blanchard SC (2018). A partially-open inward-facing intermediate conformation of LeuT is associated with Na(+) release and substrate transport. Nat Commun.

[81] Singh SK, Piscitelli CL, Yamashita A, Gouaux E (2008). A competitive inhibitor traps LeuT in an open-to-out conformation. Science.

[82] Wang H, Goehring A, Wang KH, Penmatsa A, Ressler R, Gouaux E (2013). Structural basis for action by diverse antidepressants on biogenic amine transporters. Nature.

[83] Wang KH, Penmatsa A, Gouaux E (2015). Neurotransmitter and psychostimulant recognition by the dopamine transporter. Nature.

[84] Topiol S, Bang-Andersen B, Sanchez C, Bøgesø KP (2016). Exploration of insights, opportunities and caveats provided by the X-ray structures of hSERT. Bioorganic Med Chem Lett.

[85] Boudko DY, Kohn AB, Meleshkevitch EA, Dasher MK, Seron TJ, Stevens BR, Harvey WR (2005). Ancestry and progeny of nutrient amino acid transporters. Proc Natl Acad Sci USA.

[86] Boudko DY (2012). Molecular basis of essential amino acid transport from studies of insect nutrient amino acid transporters of the SLC6 family (NAT-SLC6). J Insect Physiol.

[87] Letunic I, Bork P (2019). Interactive tree of life (iTOL) v4: recent updates and new developments. Nucleic Acids Res.

[88] Sacchi VFW (1996). Amino acid absorption biology of the insect Midgut.

[89] Sacchi VF, Castagna M, Trotti D, Shayakul C, Hediger MA (2001). Neutral amino acid absorption in the midgut of *Lepidopteran* larvae advances in insect physiology.

[90] Castagna M, Shayakul C, Trotti D, Sacchi VF, Harvey WR, Hediger MA (1997). Molecular characteristics of mammalian and insect amino acid transporters: implications for amino acid homeostasis. J Exp Biol.

[91] Giordana B, Sacchi VF, Hanozet GM (1982). Intestinal amino acid absorption in *Lepidopteran* larvae. Biochim Biophys Acta.

[92] Dow JA (1984). Extremely high pH in biological systems: a model for carbonate transport. Am J Physiol.

[93] Anderson E, Harvey WR (1966). Active transport by the cecropia midgut. II. Fine structure of the midgut epithelium. J Cell Biol.

[94] Cioffi M (1979). The morphology and fine structure of the larval midgut of a moth (*Manduca*
*sexta*) in relation to active ion transport. Tissue Cell.

[95] Wieczorek H, Putzenlechner M, Zeiske W, Klein U (1991). A vacuolar-type proton pump energizes K+/H+ antiport in an animal plasma membrane. J Biol Chem.

[96] Giordana B, Leonardi MG, Casartelli M, Consonni P, Parenti P (1998). K^+^-neutral amino acid symport of *Bombyx mori* larval midgut: a system operative in extreme conditions. Am J Physiol.

[97] Hanozet GM, Sacchi VF, Nedergaard S, Bonfanti P, Magagnin S, Giordana B (1992). The K^+^-driven amino acid cotrasporter of the larval midgut of lepidoptera: is Na^+^ an alternate substrate. J Exp Biol.

[98] Dow JAT, Peacock JM (1989). Microlectrode evidence for the electrical isolation of goblet cell cavities in *Manduca sexta* middle midgut. J Exp Biol.

[99] Castagna M, Shayakul C, Trotti D, Sacchi VF, Harvey WR, Hediger MA (1998). Cloning and characterization of a potassium-coupled amino acid transporter. Proc Natl Acad Sci U S A.

[100] Vincenti S, Castagna M, Peres A, Sacchi VF (2000). Substrate selectivity and pH dependence of KAAT1 expressed in *Xenopus*
*laevis* oocytes. J Membr Biol.

[101] Peres A, Binda F, Bossi E (2000). Effects of pH on the uncoupled, coupled and presteady-state currents generated by the amino acid cotransporter KAAT1 expressed in *Xenopus*
*laevis* oocytes. Pflugers Archiv-Eur J Physiol.

[102] Mari SA, Soragna A, Castagna M, Bossi E, Peres A, Sacchi VF (2004). Aspartate 338 contributes to the cationic specificity and to driver-amino acid coupling in the insect cotransporter KAAT1. Cell Mol Life Sci.

[103] Soragna A, Mari SA, Pisani R, Peres A, Castagna M, Sacchi VF, Bossi E (2004). Structural domains involved in substrate selectivity in two neutral amino acid transporters. Am J Physiol Cell Physiol.

[104] Bette S, Castagna M, Bossi E, Peres A, Sacchi VF (2008). The SLC6/NSS family members KAAT1 and CAATCH1 have weak chloride dependence. Channels (Austin).

[105] Bossi E, Centinaio E, Castagna M, Giovannardi S, Vincenti S, Sacchi VF, Peres A (1999). Ion binding and permeation through the *Lepidopteran* amino acid transporter KAAT1 expressed in *Xenopus* oocytes. J Physiol.

[106] Bossi E, Sacchi VF, Peres A (1999). Ionic selectivity of the coupled and uncoupled currents carried by the amino acid transporter KAAT1. Pflugers Arch.

[107] Liu ZL, Stevens BR, Feldman DH, Hediger MA, Harvey WR (2003). K+ amino acid transporter KAAT1 mutant Y147F has increased transport activity and altered substrate selectivity. J Exp Biol.

[108] Harvey WR, Wieczorek H (1997). Animal plasma membrane energization by chemiosmotic H+ V-ATPases. J Exp Biol.

[109] Mager S, Naeve J, Quick M, Labarca C, Davidson N, Lester HA (1993). Steady states, charge movements, and rates for a cloned GABA transporter expressed in *Xenopus* oocytes. Neuron.

[110] Feldman DH, Harvey WR, Stevens BR (2000). A novel electrogenic amino acid transporter is activated by K^+^ or Na^+^, is alkaline pH-dependent, and is Cl^–^ independent. J Biol Chem.

[111] Quick M, Stevens BR (2001). Amino acid transporter CAATCH1 is also an amino acid-gated cation channel. J Biol Chem.

[112] Forlani G, Bossi E, Ghirardelli R, Giovannardi S, Binda F, Bonadiman L, Ielmini L, Peres A (2001). Mutation K448E in the external loop 5 of rat GABA transporter rGAT1 induces pH sensitivity and alters substrate interactions. J Physiol.

[113] Sacchi VF, Castagna M, Mari SA, Perego C, Bossi E, Peres A (2003). Glutamate 59 is critical for transport function of the amino acid cotransporter KAAT1. Am J Physiol Cell Physiol.

[114] Mari SA, Soragna A, Castagna M, Santacroce M, Perego C, Bossi E, Peres A, Sacchi VF (2006). Role of the conserved glutamine 291 in the rat gamma-aminobutyric acid transporter rGAT-1. Cell Mol Life Sci.

[115] Bossi E, Soragna A, Miszner A, Giovannardi S, Frangione V, Peres A (2007). Oligomeric structure of the neutral amino acid transporters KAAT1 and CAATCH1. Am J Physiol Cell Physiol.

[116] Castagna M, Soragna A, Mari SA, Santacroce M, Bette S, Mandela PG, Rudnick G, Peres A, Sacchi VF (2007). Interaction between lysine 102 and aspartate 338 in the insect amino acid cotransporter KAAT1. Am J Physiol Cell Physiol.

[117] Miszner A, Peres A, Castagna M, Bette S, Giovannardi S, Cherubino F, Bossi E (2007). Structural and functional basis of amino acid specificity in the invertebrate cotransporter KAAT1. J Physiol.

[118] Castagna M, Bossi E, Sacchi VF (2009). Molecular physiology of the insect K-activated amino acid transporter 1 (KAAT1) and cation-anion activated amino acid transporter/channel 1 (CAATCH1) in the light of the structure of the homologous protein LeuT. Insect Mol Biol.

[119] Giovanola M, D'Antoni F, Santacroce M, Mari SA, Cherubino F, Bossi E, Sacchi VF, Castagna M (2012). Role of a conserved glycine triplet in the NSS amino acid transporter KAAT1. Biochim Biophys Acta.

[120] Vollero A, Imperiali FG, Cinquetti R, Margheritis E, Peres A, Bossi E (2016). The D-amino acid transport by the invertebrate SLC6 transporters KAAT1 and CAATCH1 from *Manduca*
*sexta*. Physiol Rep.

[121] Giovanola M, Vollero A, Cinquetti R, Bossi E, Forrest LR, Di Cairano ES, Castagna M (2018). Threonine 67 is a key component in the coupling of the NSS amino acid transporter KAAT1. Biochim Biophys Acta Biomembr.

[122] Peres A, Vollero A, Margheritis E, D'Antoni F, Bossi E (2012). An inverse relationship links temperature and substrate apparent affinity in the ion-coupled cotransporters rGAT1 and KAAT1. Int J Mol Sci.

[123] Zhao Y, Quick M, Shi L, Mehler EL, Weinstein H, Javitch JA (2010). Substrate-dependent proton antiport in neurotransmitter: sodium symporters. Nat Chem Biol.

[124] Cao Y, Li M, Mager S, Lester HA (1998). Amino acid residues that control pH modulation of transport-associated current in mammalian serotonin transporters. J Neurosci.

[125] Cao Y, Mager S, Lester HA (1997). H^+^ permeation and pH regulation at a mammalian serotonin transporter. J Neurosci.

[126] Peres A, Bossi E (2000). Effects of pH on the uncoupled, coupled and pre-steady-state currents at the amino acid transporter KAAT1 expressed in *Xenopus* oocytes. J Physiol.

[127] Beuming T, Shi L, Javitch JA, Weinstein H (2006). A comprehensive structure-based alignment of prokaryotic and eukaryotic neurotransmitter/Na^+^ symporters (NSS) aids in the use of the LeuT structure to probe NSS structure and function. Mol Pharmacol.

[128] Cherubino F, Miszner A, Renna MD, Sangaletti R, Giovannardi S, Bossi E (2009). GABA transporter lysine 448: a key residue for tricyclic antidepressants interaction. Cell Mol Life Sci.

[129] Bossi E, Giovannardi S, Binda F, Forlani G, Peres A (2002). Role of anion-cation interactions on the pre-steady-state currents of the rat Na(+)-Cl(−)-dependent GABA cotransporter rGAT1. J Physiol.

[130] O’mara M, Oakley A, Broer S (2006). Mechanism and putative structure of B(0)-like neutral amino acid transporters. J Membr Biol.

[131] Camargo SM, Makrides V, Virkki LV, Forster IC, Verrey F (2005). Steady-state kinetic characterization of the mouse B(0)AT1 sodium-dependent neutral amino acid transporter. Pflugers Arch.

[132] Bohmer C, Broer A, Munzinger M, Kowalczuk S, Rasko JE, Lang F, Broer S (2005). Characterization of mouse amino acid transporter B0AT1 (slc6a19). Biochem J.

[133] Margheritis E, Terova G, Oyadeyi AS, Renna MD, Cinquetti R, Peres A, Bossi E (2013). Characterization of the transport of lysine-containing dipeptides by PepT1 *Orthologs* expressed in *Xenopus*
*laevis* oocytes. Comp Biochem Physiol A Mol Integr Physiol.

[134] Bröer A, Tietze N, Kowalczuk S, Chubb S, Munzinger M, Bak LK, Bröer S (2006). The orphan transporter v7–3 (slc6a15) is a Na+-dependent neutral amino acid transporter (B0AT2). Biochem J.

[135] Margheritis E, Terova G, Cinquetti R, Peres A, Bossi E (2013). Functional properties of a newly cloned fish ortholog of the neutral amino acid transporter B0AT1 (SLC6A19). Comp Biochem Physiol A Mol Integr Physiol.

[136] Meleshkevitch EA, Voronov DA, Miller MM, Penneda M, Fox JM, Metzler R, Boudko DY (2013). A novel eukaryotic Na(+) methionine selective symporter is essential for mosquito development. Insect Biochem Mol Biol.

[137] Crooks GE, Hon G, Chandonia JM, Brenner SE (2004). WebLogo: a sequence logo generator. Genome Res.

[138] Sitte HH, Freissmuth M (2003). Oligomer formation by Na^+^-Cl^–^coupled neurotransmitter transporter. Eur J Pharmacol.

[139] Korkhov VM, Farhan H, Freissmuth M, Sitte HH (2004). Oligomerization of the γ-aminobutyric acid transporter-1 is driven by an interplay of polar and hydrophobic interactions in transmembrane helix II. J Biol Chem.

[140] Sitte HH, Farhan H, Javitch JA (2004). Sodium-dependent neurotrasmitter transporters: oligomerization as a determinant of transporter function and trafficking. Mol Interv.

[141] Jayaraman K, Das AK, Luethi D, Szöllősi D, Schütz GJ, Reith MEA, Sitte HH, Stockner T (2020). SLC6 transporter oligomerization. J Neurochem.

[142] Bartholomaus I, Milan-Lobo L, Nicke A, Dutertre S, Hastrup H, Jha A, Gether U, Sitte HH, Betz H, Eulenburg V (2008). Glycine transporter dimers: evidence for occurrence in the plasma membrane. J Biol Chem.

[143] Vandenberg RJ, Shaddick K, Ju P (2007). Molecular basis for substrate discrimination by glycine transporters. J Biol Chem.

[144] Miller MM, Popova LB, Meleshkevitch EA, Tran PV, Boudko DY (2008). The invertebrate B(0) system transporter, D. melanogaster NAT1, has unique d-amino acid affinity and mediates gut and brain functions. Insect Biochem Mol Biol.

[145] Fairweather SJ, Okada S, Gauthier-Coles G, Javed K, Bröer A, Bröer S (2021). A GC-MS/single-cell method to evaluate membrane transporter substrate specificity and signaling. Front Mol Biosci.

[146] Gold M, Egger J, Scheidegger A, Zurbrügg C, Bruno D, Bonelli M, Tettamanti G, Casartelli M, Schmitt E, Kerkaert B, Smet J, Campenhout LV, Mathys A (2020). Estimating black soldier fly larvae biowaste conversion performance by simulation of midgut digestion. Waste Manag.

[147] Čičková H, Newton GL, Lacy RC, Kozánek M (2015). The use of fly larvae for organic waste treatment. Waste Manag.

[148] Camargo SM, Singer D, Makrides V, Huggel K, Pos KM, Wagner CA, Kuba K, Danilczyk U, Skovby F, Kleta R, Penninger JM, Verrey F (2009). Tissue-specific amino acid transporter partners ACE2 and collectrin differentially interact with hartnup mutations. Gastroenterology.

[149] Margheritis E, Imperiali FG, Cinquetti R, Vollero A, Terova G, Rimoldi S, Girardello R, Bossi E (2016). Amino acid transporter B(0)AT1 (slc6a19) and ancillary protein: impact on function. Pflugers Arch.

[150] Fairweather SJ, Broer A, Subramanian N, Tumer E, Cheng Q, Schmoll D, O'Mara ML, Broer S (2015). Molecular basis for the interaction of the mammalian amino acid transporters B0AT1 and B0AT3 with their ancillary protein collectrin. J Biol Chem.

[151] Yan R, Zhang Y, Li Y, Xia L, Guo Y, Zhou Q (2020). Structural basis for the recognition of SARS-CoV-2 by full-length human ACE2. Science.

[152] Kuba K, Imai Y, Rao S, Gao H, Guo F, Guan B, Huan Y, Yang P, Zhang Y, Deng W, Bao L, Zhang B, Liu G, Wang Z, Chappell M, Liu Y, Zheng D, Leibbrandt A, Wada T, Slutsky AS, Liu D, Qin C, Jiang C, Penninger JM (2005). A crucial role of angiotensin converting enzyme 2 (ACE2) in SARS coronavirus–induced lung injury. Nat Med.

[153] Fairweather SJ, Broer A, O'Mara ML, Broer S (2012). Intestinal peptidases form functional complexes with neutral amino acid transporter B0AT1. Biochem J.

[154] Jando J, Camargo SMR, Herzog B, Verrey F (2017). Expression and regulation of the neutral amino acid transporter B0AT1 in rat small intestine. PLoS ONE.

